# Contamination Pathways can Be Traced along the Poultry Processing Chain by Whole Genome Sequencing of *Listeria innocua*

**DOI:** 10.3390/microorganisms8030414

**Published:** 2020-03-14

**Authors:** Mayada Gwida, Stefanie Lüth, Maged El-Ashker, Amira Zakaria, Fatma El-Gohary, Mona Elsayed, Sylvia Kleta, Sascha Al Dahouk

**Affiliations:** 1 Department of Hygiene and Zoonoses, Faculty of Veterinary Medicine, Mansoura University, Mansoura 35516, Egypt; mayada.gwida@gmail.com (M.G.);; 2 Department of Biological Safety, German Federal Institute for Risk Assessment, Max-Dohrn-Straße 8-10, 10589 Berlin, Germany; 3 Institute for Biology, Freie Universität Berlin, Königin-Luise-Straße 1-3, 14195 Berlin, Germany; 4 Department of Internal Medicine, Infectious and Fish Diseases, Faculty of Veterinary Medicine, Mansoura University, Mansoura 35516, Egypt; 5 Department of Food Hygiene and Control, Faculty of Veterinary Medicine, Mansoura University, Mansoura 35516, Egypt; 6 RWTH Aachen University Hospital, Pauwelsstraße 30, 52074 Aachen, Germany

**Keywords:** *Listeria innocua*, poultry production, food safety, whole-genome sequencing, single nucleotide polymorphism, listeriosis, *Listeria monocytogenes*

## Abstract

Foodborne infection with *Listeria* causes potentially life-threatening disease listeriosis. *Listeria monocytogenes* is widely recognized as the only species of public health concern, and the closely related species *Listeria innocua* is commonly used by the food industry as an indicator to identify environmental conditions that allow for presence, growth, and persistence of *Listeria* spp. in general. In our study, we analyze the occurrence of *Listeria* spp. in a farm-to-fork approach in a poultry production chain in Egypt and identify bacterial entry gates and transmission systems. Prevalence of *Listeria innocua* at the three production stages (farm, slaughterhouse, food products) ranged from 11% to 28%. The pathogenic species *Listeria monocytogenes* was not detected, and *Listeria innocua* strains under study did not show genetic virulence determinants. However, the close genetic relatedness of *Listeria innocua* isolates (maximum 63 SNP differences) indicated cross-contamination between all stages from farm to final food product. Based on these results, chicken can be seen as a natural source of *Listeria*. Last but not least, sanitary measures during production should be reassessed to prevent bacterial contamination from entering the food chain and to consequently prevent human listeriosis infections. For this purpose, surveillance must not be restricted to pathogenic species.

## 1. Introduction

Within the bacterial genus *Listeria, Listeria (L.) monocytogenes*, the causative agent of listeriosis, is widely recognized as the only species of public health concern. In humans, the clinical picture of listeriosis varies from mild to life-threatening illness with a mortality rate of 20% to 30% on average [1]. Severe cases appear mainly in vulnerable populations like pregnant women, newborns, immunocompromised, or older people [2]. The majority of listeriosis cases are foodborne [1]. Although closely related to *L. monocytogenes* (perfect synteny of genome organization)*,* the species *L. innocua* is considered as non-pathogenic because it lacks the typical virulence genes [3]. However, rare atypical hemolytic *L. innocua* strains have been described [4,5] and proven to be virulent in in vivo assays using mouse or zebrafish models [6]. Furthermore, *L. innocua* was isolated from two human patients suffering from fatal sepsis [7] and acute meningitis [8].

*Listeria monocytogenes,* as well as *L. innocua,* are widespread in natural environments such as soil, surface water, sewage, or feces of mammals and birds [9,10]. Livestock animals like poultry can be asymptomatic carriers of *Listeria* spp., and thereby lead to the unnoticed entry of the bacteria into the food chain via contaminated raw animal products [11,12]. Due to its undemanding nature and high adaptability, *Listeria* is able to persist in food production plants, which may lead to continuous contamination of food through contact with previously contaminated surfaces [13]. Raw animal products are either primarily contaminated or cross-contaminated during food-processing [12]. In this way, *Listeria* can finally end up in the food chain, posing a health threat for consumers. Detailed knowledge of entry gates and transmission routes is, therefore, indispensable to prevent food contamination and human listeriosis cases.

Because of the high similarity of *Listeria* species in terms of distribution and adaptability, the transmission path identified for one species is assumed to be transferable to other species. Hence, *L. innocua* is commonly used by the food industry as an indicator to identify environmental conditions that allow for the presence, growth, and persistence of the relevant human pathogen *L. monocytogenes* [9,14]. Using *L. innocua* as a model for *Listeria* contamination in general can help to improve surveillance and hygiene measures, which will consequently prevent human infections. However, systematic data on the prevalence of *Listeria* spp. in poultry slaughterhouses are still limited. In our study, we therefore analyze the occurrence of *Listeria* spp. in a farm-to-fork approach from the primary production stage to the final food product to reveal bacterial transmission routes. To trace *Listeria* spp. along the food chain, we applied whole-genome sequencing techniques.

## 2. Materials and Methods

### 2.1. Sample Collection

In total, 210 samples were collected from a single commercial poultry farm with five chicken flocks in separate henhouses and an affiliated slaughterhouse in 2017. The farm and slaughterhouse were located in Dakahlia Governorate, Egypt. The farm owners were asked to sign consent forms after being informed about the aims and goals of our research project and the potential health risks associated with the contamination of food products with *Listeria* spp. The study followed the ethical guidelines of Mansoura University and was approved by the responsible ethics committee (Code No. R/15).

Samples were collected from three different sources along the food production chain: on the farm, in the slaughterhouse, and from the final chicken products. Two weeks before slaughtering, 25 samples were collected on the chicken farm, all on the same day in November 2017. For soiled litter (containing fresh fecal droppings), poultry feed, drinking water, and the walls of the henhouses, samples were pooled per sampling site (*n* = 5) and per flock (*n* = 5). Five samples (20 g each) were collected from the top few centimeters of soiled litter in different locations of every henhouse (close to drinking troughs and feeding stations, from walls and near the center) and then mixed to form a composite sample of 100 g for the specific flock. The poultry feed samples were taken from the five different feeding stations of each henhouse. The water samples (20 mL each) were collected from five different drinking troughs of each henhouse and pooled to yield a composite sample of 100 mL. Farm walls were swabbed on five different sites inside each henhouse using a sterile template of 25 cm^2^ and samples from workers’ hands were collected from five individual workers. In the slaughterhouse, 13 cloacal swabs were collected from each of the five flocks just before slaughter (*n* = 65). The birds were randomly selected. In addition, 20 surface swabs (10 from slaughterhouse walls, 5 from tables and 5 from knives) were taken during processing. Another 100 samples were taken after slaughter, including swabs from 80 whole carcasses, 10 chicken fillets, and 10 livers.

Hand swabs (palm, between fingers, fingertips, and fingernails) were essentially carried out according to the protocol of Genigeorgis and colleagues [15]. We used buffered peptone water (BPW; Oxoid, Basingstoke, UK) to moisten the cotton swabs and as enrichment broth. Soiled litter, poultry feed, and drinking water were sampled following standard procedures using sterilized spatulas or syringes [16,17]. The samples were homogenized with a stomacher and stored in sterile bags at 4 to 8 °C until transport to the laboratory. Swabs from surfaces of walls and tables in the slaughterhouse were collected according to the guidelines of the American Public Health Association [18]. Briefly, four 100 cm^2^ regions of the sampling site were swabbed with sterile sponges moistened with 40 mL of BPW in several horizontal and vertical movements. The sponges were then transferred to sterile bags containing 160 mL of BPW to yield a final volume of 200 mL. The farm walls were swabbed using the same technique. The two sides of the butcher’s knives were swabbed with BPW-moistened cotton swabs instead of sterile sponges. Cloacal swabs (from the mucosal wall) were collected from living chicken with sterile cotton-tipped swabs pre-moistened in BPW. Swabs from chicken carcasses were collected after evisceration using the method described by McEvoy and colleagues [19]. Briefly, each swab was moistened just before use with 25 mL of BPW and put into a sterile plastic bag after sampling. Chicken fillet and liver samples (~25 g each) were sliced with a sterile scalpel and put into a sterile stomacher bag. All samples were processed under aseptic conditions and then directly sent to the laboratory for further analyses.

### 2.2. Listeria Isolation and Identification

*Listeria* spp. were isolated and identified as described in the Bacteriological Analytical Manual of the U.S. Food and Drug Administration [20]. For solid samples (poultry feed, chicken fillet, and liver) and water samples, 25 g or ml were added to 225 mL *Listeria* enrichment broth without antibiotic supplement, pH 8.6 (Oxoid) and homogenized in a stomacher for two minutes. The swab samples were transferred to 10 mL *Listeria* enrichment broth. Homogenates of solid samples, water samples, and swabs were incubated at 30 °C for 4 h. Then, *Listeria* selective enrichment supplements (Oxoid), including nalidixic acid, cycloheximide, and acriflavine, were added and the broth cultures were incubated at the same temperature for another 24 to 48 h. An inoculation loop of the enriched sample was incubated on Oxford agar (Oxoid) at 35 °C for 24 to 48 h. At least five colonies showing a black halo characteristic for *Listeria* spp. were picked, transferred onto tryptic soy agar plates with 0.6% yeast extract and incubated at 30 °C for 24 to 48 h. These presumptive *Listeria* isolates were stored at –80 °C in brain heart infusion with 20% glycerol.

### 2.3. Matrix-Assisted Laser Desorption/Ionisation Time-of-Flight (MALDI-TOF) Mass Spectrometry

After thawing, the bacteria were plated onto sheep blood agar and incubated overnight at 37 °C. Mass spectrometry samples were prepared using the direct smear method [21]. Species identification was performed using the MALDI Biotyper^®^ Subtyping Module (Bruker Daltonik, Bremen, Germany) according to the manufacturer’s instructions.

### 2.4. Genomic DNA Extraction and Next Generation Sequencing

All strains identified as *Listeria* spp. by MALDI–TOF MS were again grown on sheep blood agar overnight at 37 °C. Bacterial cells were harvested and lysed following the Pulse Net standardized laboratory protocol for whole-genome sequencing of Gram-positive bacteria (https://www.cdc.gov/pulsenet/pdf/pnl32-miseq-nextera-xt.pdf). We extracted DNA with the QIAamp DNA Mini Kit (Qiagen, Hilden, Germany) according to the manufacturer’s instructions.

Sequencing libraries were constructed using the Nextera XT Sample Preparation Kit (Illumina, Inc., San Diego, CA, USA). Sequencing was performed in paired-end mode with 2 × 300 bp reads on the Illumina MiSeq sequencer using the MiSeq Reagent v3 600-cycle Kit (Illumina).

#### 2.4.1. Multilocus Sequence Typing (MLST)

Multilocus sequences types (ST) and clonal complexes (CC) were determined according to the scheme available at https://bigsdb.pasteur.fr/listeria/listeria.html.

#### 2.4.2. Single Nucleotide Polymorphism (SNP) Analysis

Sequences were trimmed with Trimmomatic version 0.36 [22] using default parameters. Trimmed reads were mapped to the closed reference genome of *L. innocua* (NC_003212.1) in BioNumerics version 7.6 (Applied Maths, Gent, Belgium), followed by SNP calling. Strict SNP filtering with software default parameters was applied.

#### 2.4.3. In Silico Screening for Virulence Factors

Trimmed reads were de novo assembled with SPAdes version 3.11.1 [23]. Assembled genomes were used for virulence gene screening with ABRicate version 0.8 [24] using the Virulence Factor Database (VFDB) (2597 sequences, [25], last updated 9 July 2019). The *L. monocytogenes* reference strain EGDe (NC_003210.1) was included in the screening as a representative for a pathogenic strain. A cut-off of at least 80% gene identity was applied for gene presence.

#### 2.4.4. Data Storage

The data for this study have been deposited in the European Nucleotide Archive (ENA) at EMBL-EBI under accession number PRJEB36384.

## 3. Results

### 3.1. Prevalence of Listeria spp. along the Poultry Production Chain

*Listeria innocua* was the only *Listeria* species identified by MALDI–TOF MS and was isolated from 17% (36/210) of the samples along the poultry production chain (Table 1). The prevalence of *L*. *innocua* on the farm level was 28% (7/25). *Listeria innocua* was found in 100% (5/5) of the swabs from farm walls and in 20% (1/5) of the samples from soiled litter or poultry feed. Both workers’ hands and drinking water tested negative for *L. innocua*. A total of 9 out of 85 samples (11%) collected in the slaughterhouse revealed *L*. *innocua*. Tables and abattoir walls were contaminated, with 40% (2/5) and 20% (2/10) positive swabs, respectively, whereas knives were tested negative. Five out of 85 (7%) cloacal swabs taken from three of the five chicken flocks tested positive. Finally, *L. innocua* was identified in 20% (20/100) of the food samples, with 11% (9/80) of the carcasses, 50% (5/10) of raw chicken fillets, and 60% (6/10) of the liver samples being positive.

### 3.2. Genomic Analysis

**Figure 1:**
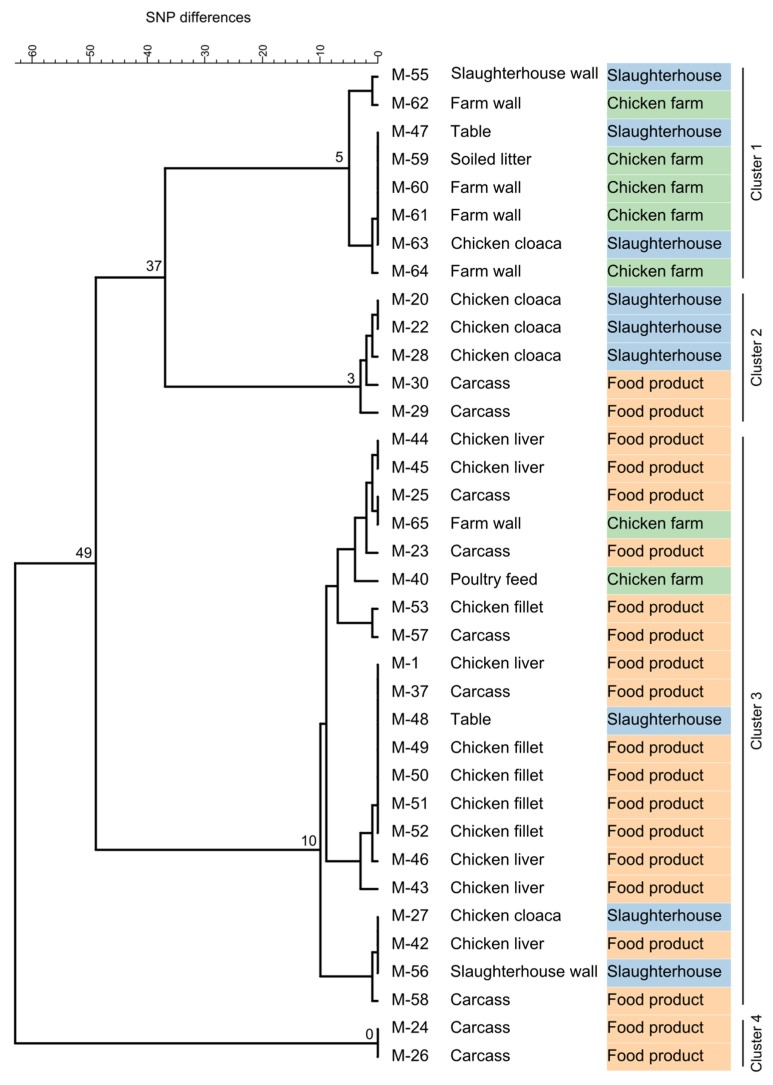
Complete linkage tree summarizing SNP analysis results from 36 *Listeria innocua* isolates. Node labels indicate the maximum SNP difference in the branch. Isolates fell into four distinct clusters differing by no more than 5, 3, 10, or 0 SNPs. Clusters were not restricted to a specific sampling site (first column) or sampling stage (second column) except for Cluster 4.

**Figure 2:**
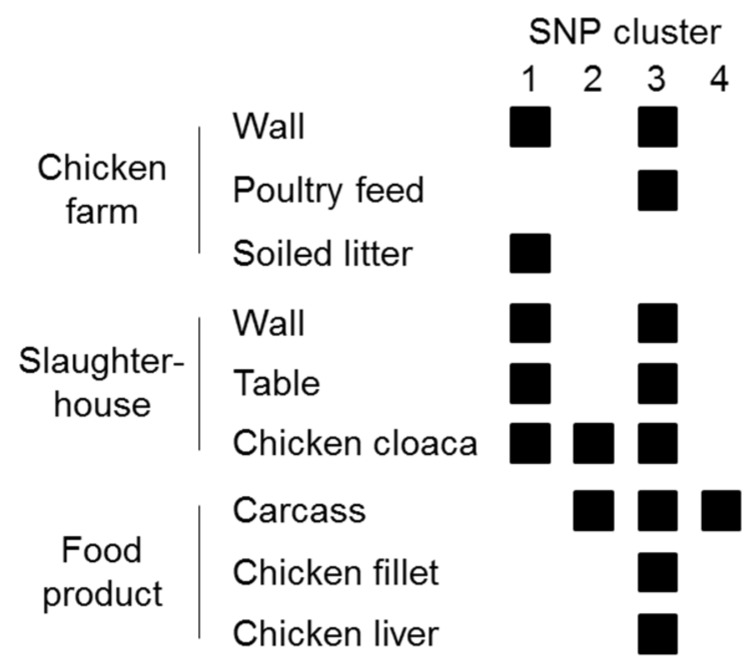
Visualization of contaminated sites and presumable transmission routes based on SNP clusters. Cross-contamination is likely to have happened between all production stages.

Whole-genome sequencing of the 36 *Listeria* isolates confirmed the MALDI–TOF MS results and clearly assigned them to the species *L. innocua* (94% to 100% of reads mapping to the *L. innocua* Clip11262 complete genome, NC_003212.1). All isolates belonged to the same *L. innocua*-specific MLST ST 530 (corresponding to CC ST530, Lineage: *L. innocua*). Sequencing coverage ranged between 43- and 132-fold (median 78). The 36 isolates showed 0 to 63 SNPs difference (median 41) and formed four distinct clusters of isolates differing by no more than 0, 3, 5, or 10 SNPs (Figure 1). Clusters were not restricted to a specific sampling site or sampling stage except for one cluster (no. 4), which included two isolates from carcasses (Figure 1 and Figure 2).

**Figure 3:**
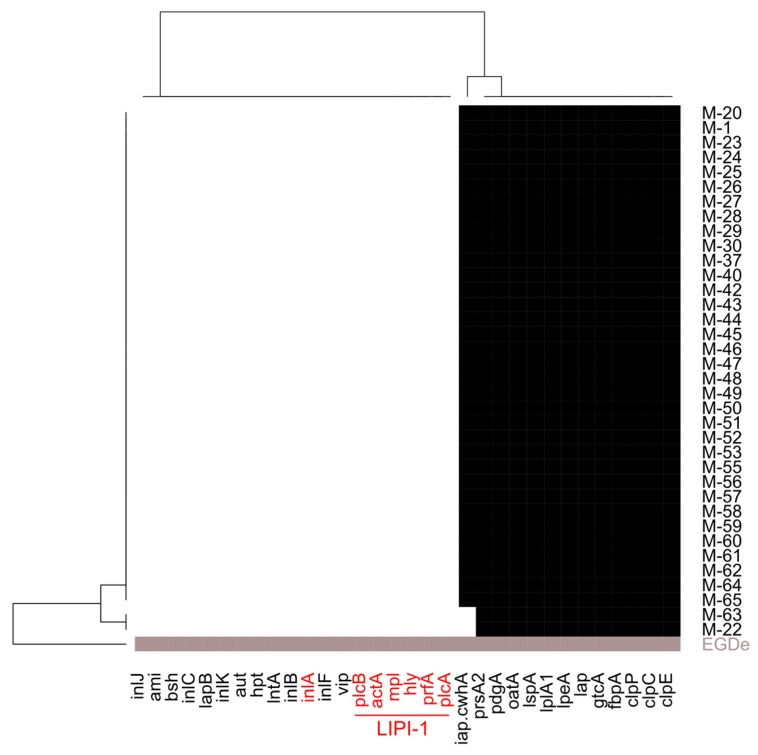
Heatmap of in silico detected virulence genes in the *Listeria (L.) innocua* study population (black: gene present; white: gene absent) compared to the *L. monocytogenes* reference strain EGDe (grey: gene present). None of the *L. innocua* study isolates contained virulence factors like *inlA* or the *Listeria* pathogenicity island 1 (LIPI-1; in red) that are found in atypical hemolytic *L. innocua* [6].

The *L. innocua* isolates under study harbored 12 to 13 *L. monocytogenes* virulence genes (Figure 3). However, the *Listeria* pathogenicity island LIPI-1 and internalins, especially *inlA*, which are genetic determinants for virulence in either atypical hemolytic *L. innocua* strains or in the pathogenic species *L. monocytogenes* [6], were only found in the sequence of the *L. monocytogenes* reference strain EGDe and were missing in all *L. innocua* isolates.

## 4. Discussion

At the three stages of the poultry production chain investigated (farm, slaughterhouse, food products), the prevalence of *L. innocua* ranged from 11% to 28%.

As we only looked into one specific farm-to-fork continuum (one farm, one slaughterhouse) in our study, the prevalences found at the various production stages cannot necessarily be generalized. However, our prevalence rates on farm level were in agreement with a previous study on a poultry farm in Egypt [26] where *L*. *innocua* was found in 20% (4/20) of samples from poultry feed but not in drinking water (0/20). However, *L*. *innocua* was more prevalent in soiled litter than in our study (35% (28/80) vs. 20% (1/5)). The observed difference could be explained either by the very different sample sizes or by variable efficiency of biosecurity practices on the farms. In our study, the overall prevalence of *L. innocua* on the farm was 28%. The prevalence reported for *Listeria* spp. (especially *L. monocytogenes* and *L. innocua*) on farms in various countries varied widely, ranging from 1.4% to 53% [9,27,28,29,30,31]. Accordingly, the detection rates for *Listeria* spp. in soiled litter (10%–53%), poultry feed (70%), drinking water (10%), soil (30%), and grass (6%–43%) were quite variable. In accordance with our results, *L. innocua* was the predominant species, representing up to 78% of the *Listeria* isolates.

The slaughterhouse prevalence of *L. innocua* in our study was 11%. The highest rate was found in swabs collected from tables (40%, 2/5), indicating that hygiene measures were not always properly applied to remove surface contamination. Lower contamination rates were found on the walls (20%, 2/10) and in cloacal swabs (8%, 5/65). A positive cloacal swab indicates intestinal colonization of the chicken. Hence, the positive rate of cloacal swabs is actually an indicator of the prevalence of carrier animals in the livestock population that form the basis for zoonotic entry of *Listeria* into the food chain. In our study, according to the microbiological results from cloacal swabs, three of the five chicken flocks tested were carriers of *L. innocua*. A lower detection rate (2%, 7/400) for *L. innocua* in cloacal swabs from laying hens was reported from Bavaria, Germany [28]. In the suburbs of Tokyo, *Listeria* spp. were found in 5% of 150 fresh fecal droppings collected on four chicken farms [32]. In a Danish study, *Listeria* spp. could not be isolated at all from 50 cloacal swabs taken at the abattoir of 71 broiler flocks [27]. Obviously, there are major differences in the *Listeria* prevalence rates in chicken livestock among countries.

In our dataset, the overall occurrence of *L. innocua* in raw poultry meat and chicken products was 20% (20/100). Higher prevalence rates for *L. innocua* in raw poultry meat were reported from Spain (66%), Turkey (58%), Italy (40%), Jordan (50%), and Egypt (31%) [26,33,34,35,36] while lower rates were reported from Morocco (14%) and Iran (19%) [37,38]. Contamination of raw animal products may occur after slaughter or during food processing. Major influencing factors are the primary prevalence of the pathogen in the livestock population, on the one hand, and hygiene measures such as surface disinfection during processing, on the other hand.

To get detailed insights into possible transmission routes along the poultry production chain, we analyzed single nucleotide polymorphisms of *L. innocua* isolates at various stages of production (Figure 1). Based on the fact that natural mutation rates in the genus *Listeria* are low, a very low number of SNP differences between two or more strains are commonly used as an indicator of their epidemiological relatedness [39,40]. The guiding assumption is that a small genetic difference between strains indicates a common origin. In our study, based on the high genetic relatedness of *L. innocua* isolates from different sampling sites from farm to final food product, transfer between all stages of the production chain appears very likely. Although no directional information can be extracted from the genetic data alone, accompanying metadata can be used to speculate about causal relationships between contaminated sites and transmission routes (Figure 2). For instance, zero SNP differences were identified between *L. innocua* isolates from chicken liver, fillet, carcass, and a slaughterhouse table (Figure 1; within Cluster 3). Presumably, the same bacterial strain has been transferred between all these sampling sites, indicating cross-contamination during the processing of the slaughtered chicken. Of positive note is the fact that all samples from knives and hands were negative for *Listeria* spp., indicating that hygienic measures are already successfully applied to a certain extent, although the relatively low number of samples taken from both sites may qualify this statement. However, knowing that there is likely entry of *L. innocua* into the food processing plant from the farm, other surfaces should be under proper internal monitoring as well. For example, the same strain (zero SNPs difference) could be found on a table in the slaughterhouse and in cloacal swabs, as well as on farm walls and in the soiled litter (Figure 1; within Cluster 1). Consequently, bacterial contamination on the farm has reached the processing level, thereby posing a risk for further cross-contamination during processing steps performed on the table. Since contamination with *L. innocua* was already present on the farm, the food operator should reassess the sanitary measures applied and the way how chickens are introduced into the processing stage to prevent contamination from entering the production chain. If no reasonable measures are taken, *Listeria* can establish persistence in food processing plants, which may form the basis for repeated re-contamination [13,41,42].

*Listeria innocua* strains isolated in our study did not show any genetic virulence determinants needed for human or animal infection, as described for *L. monocytogenes* strains and rare hemolytic *L. innocua* strains [6]. In addition, all isolates were non-hemolytic on sheep blood agar. Therefore, it is highly unlikely that they would have been able to cause human infection. Furthermore, the pathogenic species *L. monocytogenes* was not detected. However, as previously mentioned, the Oxford medium used in our study does not allow for a distinction between colonies of different *Listeria* species [43]. A chromogenic agar, as described in ISO 11290:1:2017, would have been able to improve *L. monocytogenes* detection but it was not available in Egypt at the time of the study. Therefore, presumptive *Listeria* spp. colonies were randomly selected and *L. monocytogenes* isolates could have been missed. Additionally, *L. innocua* can produce a bacteriocin-like substance against *L*. *monocytogenes* and usually grows faster in enrichment broth, leading to an underestimation of the prevalence of *L*. *monocytogenes* [27,43].

While several studies from Egypt have reported frequent contamination of foodstuffs such as meat and dairy products with different *Listeria* spp. [26,44,45,46,47] such food has not yet been associated with documented outbreaks of listeriosis. A major reason for this is probably the lack of a surveillance system for human listeriosis in Egypt and hence underreporting of cases. As a result, the real public health burden caused by *Listeria* contamination throughout the food chain is very difficult to assess. Further close monitoring of slaughtering and company hygiene practices and their continuous adjustment and improvement will help to gain insights into the risks emerging from different food sources and will make an essential contribution to prevent listeriosis cases.

## 5. Conclusions

Our study did not reveal any *L. monocytogenes* contamination, but *L. innocua* existed throughout the entire chicken meat processing chain from stable to table. Given that *L. innocua* was not only isolated from environmental samples on the farm and in the slaughterhouse but also from cloacal swabs, chicken can be seen as a natural source of *L. innocua*. The presence of any non-pathogenic *Listeria* spp. like *L. innocua* in processing lines and foodstuffs is a good indicator for poor hygienic conditions and serves as an alarming sign for the need to implement appropriate hygiene practices. Through knowing and eliminating risk factors, contamination of poultry food products with the pathogenic species *L. monocytogenes* can be effectively prevented.

## Figures and Tables

**Table 1:** Occurrence of *Listeria innocua* along the poultry production chain

Source of Sample	Number of Samples Tested	Number of Positive Samples	% of Positive Samples
**Chicken farm**	**25 ^1^**	**7**	**28**
soiled litter	5	1	20
drinking water	5	0	0
poultry feed	5	1	20
farm wall	5	5	100
workers’ hands	5 ^2^	0	0
**Slaughterhouse**	**85 ^2^**	**9**	**11**
chicken cloaca	65	5	8
slaughterhouse wall	10	2	20
knife	5	0	0
table	5	2	40
**Food product**	**100 ^2^**	**20**	**20**
carcass	80	9	11
chicken fillet	10	5	50
chicken liver	10	6	60
**total**	**210**	**36**	**17**

^1^ pooled samples; ^2^ individual samples.
